# Distributive Leadership Within an Emerging Network of Integrated Youth Health Centres: A Case Study of Foundry

**DOI:** 10.5334/ijic.4709

**Published:** 2020-12-01

**Authors:** Amy Salmon, Saranee Fernando, Mai Berger, Karen Tee, Kristy Gerty, Warren Helfrich, Pamela Liversidge

**Affiliations:** 1 Centre for Health Evaluation and Outcome Sciences, The School of Population and Public Health, University of British Columbia, Vancouver, BC, CA; 2 Foundry Central Office, Vancouver, BC, CA

**Keywords:** integrative care, distributive leadership, mental health, adolescent health, system transformation

## Abstract

**Background:**

Distributive leadership has been proposed as an effective means towards achieving integrated health services. This study draws from the case of *Foundry*, a network of integrated youth health centres in British Columbia, Canada, and explores the function and impact of distributive leadership in the context of a large-scale effort towards integrated service delivery for youth experiencing mental health and substance use challenges.

**Methods:**

Qualitative data was obtained from a developmental evaluation of Foundry using a longitudinal, ethnographic approach. Over 150 participants involved in the development of six Foundry centres were interviewed individually or in focus groups. Purposive and theoretical sampling strategies were used to maximize the diversity of perspectives represented in the data set.

**Results and Discussion:**

Distributive leadership was observed to be a facilitator for achieving service and system-level integration. Distributive leadership was effective in promoting streamlined service provision, and coordinating efforts towards optimized access to care. A new culture of leadership emerged through collaboration and relationship-building based on a common value system to prioritize youth needs.

**Conclusion:**

As Foundry, and other integrated youth services, continues to expand, distributive leadership shows promise in assuring diverse and coordinated input for integrating services.

## Introduction

Leading change to transform health and social care systems requires the development of increasingly larger networks of diverse groups of leaders working interdependently towards a shared vision [1]. The World Health Organization proposes that distributive leadership between multiple actors who work together across professional and organizational boundaries is a way to achieve people-centred and integrated health services [2,3]. ‘Distributive leadership’ is an approach involving concertive action achieved by spontaneous collaboration through intuitive working relationships [4,5]. These relationships may evolve organically over time, or by institutionalised practices [4,6]. Currie and Lockett (2011) describe distributed leadership as a form of “shared leadership” [7,8,9] and as “a group phenomenon, with followers playing a role in influencing and creating leadership” [7,9]. While seen to be an advantageous approach for promoting change, there is a dearth of scholarship examining how distributive leadership as a construct is practiced, and with what results, within complex health systems.

The urgency of need for health and social care system transformation and for promoting meaningful and effective integrated care delivery is particularly apparent in the area of youth mental health and substance use care [10]. Most mental disorders begin during youth between 12 and 24 years of age [11]. Recent studies have shown a significant and persistent gap between the identified service needs of young people living with mental health concerns and their abilities to access services. In some Canadian jurisdictions, as few as 25% of youth with diagnosable mental health and substance use disorders receive the services they need [12]. This gap is not due to a lack of evidence-based treatments. Rather, the challenge in most jurisdictions lies in the creation of low-barrier access points that make these treatments readily available, affordable, coordinated, and acceptable, and which support young people to engage in evidence-based interventions as they transition from adolescence to adulthood [13,14].

The need for integrated approaches to youth mental health and substance use concerns is underscored by the combination of individual and systemic factors that frequently combine to frustrate efforts to help young people receive timely, appropriate, and effective supports. The “siloing” of mental health services often means that co-occurring health concerns, and issues related to social determinants of health are addressed through separate and often duplicative care protocols and pathways. Lack of mental health literacy and persisting high levels of stigma can discourage young people from discussing mental health, substance use, or sexual health concerns with their regular or usual health care provider, and push young people toward walk-in clinics, where often no continuity of care exists and counselling is limited [15,16].

Within the context of mental health and youth development, experts recommend the enhancement of care systems through integration, consideration of context, and inclusion of individual voice [17,18,19,20,21]. Internationally, responses to the challenge favour lower barrier, “one stop shops” that provide integrated health and social services in youth-friendly environments [22,23,24,25]. One such initiative currently underway in British Columbia, Canada is *Foundry*: a health care movement whose goal is to transform access to care for young people ages 12–24. In addition to offering primary care, social services, peer support, and system navigation assistance, Foundry’s integrated care centres offer mental health and substance use services (www.foundrybc.ca). These centres were built on partnerships between several government, philanthropic partners, health authority, and non-profit organizations to deliver the health and social services needed by young people in one accessible location. Operationally, the impetus for the Foundry movement was to dismantle the salient problem of siloed health care delivery through integrating health care services to streamline care trajectories for youth. Therefore, instead of modifying health services themselves, Foundry restructures the health care system by reconfiguring how services are delivered through integration.

The Foundry movement includes two major phases of integration occurring at both the systems and service delivery level. Foundry centres present with differing levels of integration based on unique geographic, demographic, and service delivery contexts of each community, as well as their specific local challenges and strengths. Findings from the developmental evaluation of Foundry captured perspectives to inform the level at which system integration was achieved to create Foundry centres. At a later stage in the development evaluation of Foundry, the extent of service integration will become apparent through acquiring patient perspectives, where the experience of care can be learned through engaging the patient population and those who are currently accessing care through Foundry.

In this article, we will present findings from a developmental evaluation of *Foundry* which highlights the function and impact of distributive leadership in the context of a large-scale effort to achieve a platform for integrated service delivery across the youth health and social service landscape. In so doing, we identify ways in which emerging understanding of the kind of leadership that can be required to foster and embed integrated approaches to care that were identified by *Foundry* leaders aligns with distributive leadership approaches.

### Defining Distributive Leadership

Günzel-Jensen et al. 2018 and Bennet et al. 2003, have identified three primary requirements for distributive leadership:

1. Leadership is an emerging key feature of the group: Distributed leadership emphasizes leadership as an emergent quality of a group or network of interacting individuals, as opposed to leadership as a phenomenon which arises from the individual [26]. Individuals work together to pool their initiative and expertise to produce outcomes as a product or energy which is greater than the sum on their own individual actions [26].
2. There is openness towards who can perform leadership tasks, with focus on inclusion rather than exclusion: There is an ability to expand the conventional net of leaders, raising the question of which individuals and groups are to be brought into leadership or seen as contributors to it [26].
3. Leadership tasks are shared among the many, not only the appointed leaders: A concertive dynamic is created and fostered by numerous, distinct, and germane perspectives [26].

The construct of distributive leadership was not directly embedded within the evaluation of Foundry at the outset. However, as we will demonstrate, emerging understandings evident among Foundry leaders about how to lead the work of transforming access to mental health services for young people through intentional service integration aligns with these features of distributive leadership.

### Leadership at Foundry

**Figure 1:**
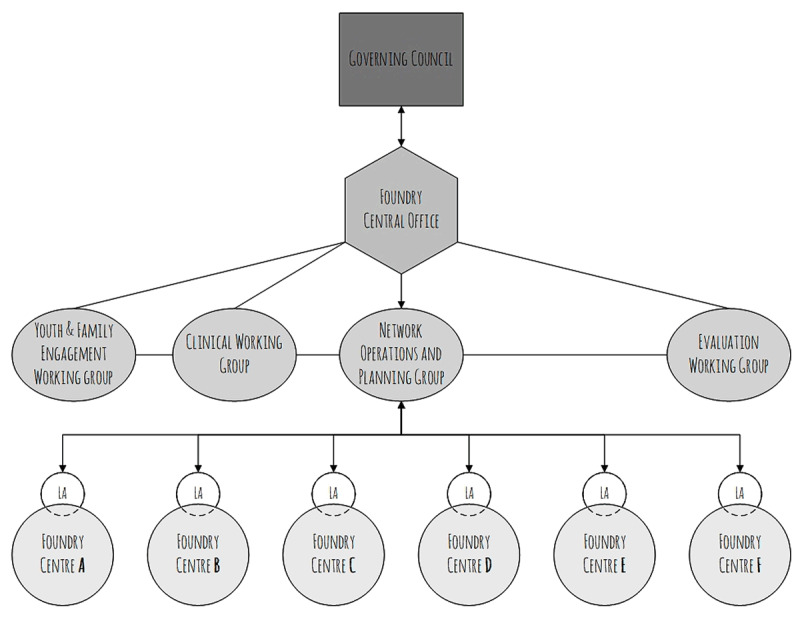
The governance structure of Foundry Central Office during the proof-of-concept period.

During Foundry’s proof-of-concept period (2016–2018), five new integrated youth wellness centres were established across BC, following a model adapted from a “prototype” centre that had opened 1 year prior. Foundry’s leadership structure, comprising a provincial Governing Council, Foundry Central Office, and Lead Agencies (LA) support the development of Foundry centres through integrating services and practices within a complex system. In addition, a number of working groups (including the Youth and Family Engagement Working Group, the Clinical Working Group, the Network Operations and Planning Group, and the Evaluation Working Group) were established by Foundry Central Office, comprised of Foundry Central Office leadership and staff, representatives of lead agencies operating each of the Foundry Centres, and other key decision-makers at the regional and provincial levels in areas where the new Foundry centres were located. See Figure 1 for a schematic of this governance structure.

**Figure 2:**
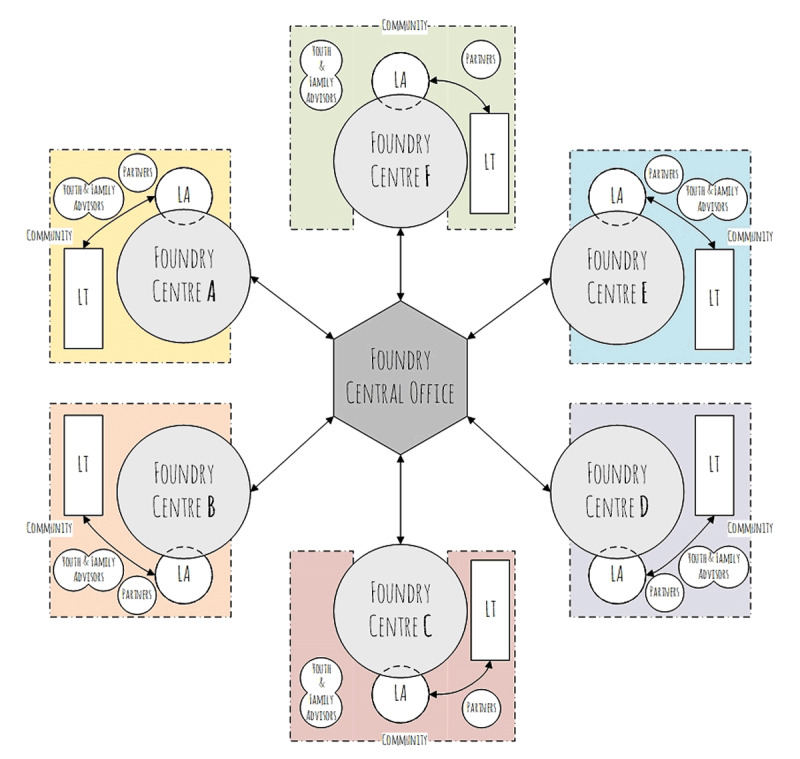
Leadership structures of the Foundry centres.

Foundry was initially conceived as a “collective impact” initiative, with the Foundry Central Office acting as its “backbone” organization [27]. It was formed to provide provincial leadership and supports, convene multi-sector partners, mobilize people and organizations toward a shared vision, build a body of knowledge to support centre development and operations, and act as a bridge between government, health, and community organizations. Lead Agencies were selected in each community to have organizational accountability for the overall financial management and service delivery accountability for their centre. However, by agreement with all partners, Lead Agencies rely heavily on direct and indirect contributions from partnering agencies to deliver all onsite services, thus requiring a coordinated and collaborative approach. Similarly, communities applying to develop a Foundry Centre during the proof of concept period were required to demonstrate how services would complement and extend, but not duplicate, existing services available to young people and families in their communities. Lead Agencies in each community developed and led inter-agency information sharing agreements and processes with local partners to support integrated care, while leading local centre establishment and working with the Foundry Central Office to develop, implement and operationalize the Foundry Service Model. With the leadership and support from a Lead Agency, Leadership Tables (LT) at each Foundry centre provided guidance to address community needs while actively consulting with youth and families sitting on advisory boards and community partners. A schematic of this structure is provided in Figure 2.

## Methods

The data reported on in this paper are drawn from work commissioned to support evidence-informed, real time decision making on the adaptive development of the Foundry model of integrated youth health service planning and delivery. This work was structured as a developmental evaluation using a longitudinal, ethnographic approach.

### Developmental evaluation using a longitudinal, ethnographic approach

Developmental evaluation is a relatively new approach that is intended to support the creation and implementation of dynamic, complex innovations. Developmental evaluation is typically used to define and refine new models and approaches at the earliest stages of innovation, when the path to achieving success is unknown and evidence regarding expected outcomes is scarce or unclear [28]. Developmental evaluation may also inform understandings of organizational learning, as it offers strategic creation, capture, and internalization of knowledge [29]. A longitudinal ethnographic design was used to structure the developmental evaluation, to support immersion and prolonged engagement with Foundry and the six Foundry centres, providing ongoing opportunities to observe the inner and outer workings of these entities, and to ask questions and clarify assumptions that were driving decision-making and experiences over time.

The evaluation was carried out between February 2016 and April 2018, and was divided into two phases. The first phase examined the formation of Foundry Central Office and its role and function. In the second phase, attention shifted to the experiential learning occurring in the first six Foundry centres related to the early operation of these centres. All aspects of the developmental evaluation were carried out in accordance with Providence Health Care’s ethical requirements for evaluation and quality improvement activities, and were assessed using ARECCI processes [30].

### Data sources

All ethnographic data was collected by the primary evaluator (AS). During the first phase of the evaluation, interviews were conducted with Foundry Central Office staff, combined with participant and non-participant observations of activities and events initiated by Foundry Central Office. Field notes recorded acts, decisions, interpretations, and processes, and were expanded with analytic memos and reflective notes. Observations at Foundry Central Office continued on a semi-weekly basis for 17 months, until thematic saturation had been achieved. Semi-structured individual and group interviews in the second phase of the developmental evaluation were conducted in three cycles. Participants were interviewed at least once and up to three times each, depending on their role at Foundry and availability. Interviews were 30–120 minutes in length. The majority of interviews took place at or near the Foundry centre, Lead Agency, or Central Office site. Telephone or videoconference interviews were conducted if face-to-face interviews were not possible.

### Participants and Sampling

Over 150 participants were interviewed individually or in focus groups over the three cycles of the second data collection phase. Purposive and theoretical sampling strategies were used to maximize the diversity of perspectives represented in the data set, with some limited use of snowball sampling. Individuals were invited to participate in individual or focus group interviews if they (or their organization) had a direct role in the development or implementation at one or more of the six Foundry centres, or if they played a key role in the establishment of Foundry Central Office or the Foundry movement. Participants included Foundry centre staff, youth and family advisors, the Foundry Central Office team, Governing Council members, Lead Agency personnel, representatives of partner agencies, and local leadership table partners.

### Data analysis procedures

With consent, all of the interviews were audio recorded and transcribed verbatim. Analyses were performed using inductive and thematic coding, informed by a modified grounded theory approach and aided by QSR International’s NVivo 11 qualitative data analysis software. Coding was competed by two independent coders (MB and SF) who had not been involved in data collection. Descriptive categories were generated from the data using a combination of open coding and a priori interests embedded in the evaluation questions. The coding process was also informed by ongoing discussions with the primary evaluator and, given developmental evaluation’s primary commitment to be utilization-focused, with key individuals in leadership and evaluation roles at Foundry Central Office. Reflective sessions were conducted at the conclusion of Cycles 1, 2, and 3 with Foundry Central Office and each of the local Foundry centre leadership tables to enrich the data and to member-check the evaluators’ interpretations.

## Results

### The Emergence of a New Culture of Leadership and Creating a Sense of “We”

From the outset of Foundry’s work at both the local and provincial levels, individuals in leadership roles quickly identified that achieving the aim of transforming access through service integration would require leaders at all levels to lead differently than they had in the past to implement policy or programs within a single service, organization, or system. The main challenge identified by Foundry leaders at the inception of this initiative was understood to be a need not only to create a single new service for young people in each participating community, but to create new mechanisms and opportunities for existing systems and services to work better together. In the words of one respondent:

“The most significant barriers have to do with – not with the lack of service, but a lack of the system of the services… For a variety of reasons, we don’t work well as a system. What is lost or who gets lost in there is kids and families. This is an opportunity to work smarter, think differently, and work together.” (Centre A, Staff)

A new culture of leadership emerged that engaged leaders in health, social services, non-profit community-based organizations, philanthropic partners, and Foundry Central Office staff. An important first step in creating this new culture of leadership was a concerted effort to dismantle hierarchies among Foundry stakeholders. As one respondent indicated:

“So that, to me, is…an important part of culture setting- that flattening of hierarchies, where we are willing to be the ones who do that.” (Centre A, Staff)

The next step taken by leaders toward a distributed leadership approach was seen in efforts to create a shared sense of ownership of, and reinforcing relationships between, Foundry and community partners. While a single Lead Agency in each community assumed legal and administrative responsibility to their local Foundry centre, leaders found that the task of creating and implementing a local integrated service delivery approach, in which multiple agencies would be involved at delivering services at the centre in a coordinated way, required all partners to cultivate an understanding that the centre would be “our Foundry”. In this way, leadership was not seen to be “the monopoly of responsibility of just one person… [but a] more collective and systemic understanding of leadership” [5]. Across accounts, a clear shift was identified in the ways respondents began to describe the work as creating a sense of unity amongst stakeholders:

“I love it here. I get a real sense of team. I actively seek out people in the building when I need them and I know that I can do that.” (Centre E, Staff)

“I think how we do it … is that we have and will continue to attend to the fact that there needs to be a “we.” This can’t just be a one organization initiative.” (Centre A, Staff)

Sharing a vision for Foundry was accomplished by bringing on invested partners, ensuring that they had a voice in the initiative, and involving those invested in placing youth needs first to reach common goals and acquire a broad understanding of the Foundry movement. As one respondent stated, “…recognizing that we’re all there in the best interest of raising the next generation and supporting our youth.” (Centre A, Partner Agency)

Local leadership tables, comprised of decision-makers from organizations and agencies who were directly involved in delivering services on-site at centres, or indirectly involved by making and receiving referrals of young people for services, played a key role in promoting this new culture of leadership. Members of the leadership tables also ensured that progress in creating Foundry involved collective decision-making informed by diverse stakeholder perspectives, as described in the following account:

“I think it would be trying to identify and problem solve areas where we could improve, especially with feedback, because they all have these little parts of perception. The elephant thing, right? I feel the trunk and someone else feels something else, so I think they all have different perspectives to offer around what’s working, what are we doing great, what isn’t so great, what are the pieces that we can maybe improve on, and get some real input and ownership that this is a collective that we’re all responsible for.” (Centre F, Staff)

At the provincial level, the clinical working group would meet regularly with community partners to prioritize tasks and do the work of Foundry while operating on short timelines. This work required inviting key players to the table with strong yet flexible leadership qualities to collectively spearhead issues based on priority:

“It’s all of our…they really are, they allow people to take key lead roles, it’s not like…again, about metaphors, it’s like the swallow migration, you see different individuals taking the leads when the need arises. It’s very fluid and they model that.” (Centre A, Staff)

At local Foundry Centres, the sense of shared purpose in operationalizing “one-stop-shops” was the undercurrent for moving towards resource and knowledge sharing. Respondents indicated benefits to resource and knowledge sharing as diversifying perspectives among partners to build a foundation for comprehensive care:

“Yes. I like the idea that we’re sort of pooling for the Foundry. Hopefully, the idea is to pool sort of our knowledge and our resources. And so, we hopefully maybe can even make it where our expertise is available for kids that maybe aren’t in the crisis, but we can support our colleagues and supporting their youth, so it doesn’t have to go to a crisis – would be one, you know, benefit, I sort of see as sort of partnering with so many other different agencies…” (Centre D, Partner Agency)

While new skills and competencies were added through shifting leadership structures including staff turnover, trust-building and sustainability of relationships would sometime erode under these circumstances. In terms of operations, staff turnover hindered planning and extended time spent on training and familiarizing with the work of Foundry. As a result, existing staff would “stretch” to satisfy their mandate with sustained and dedicated commitment to move the work forward in an ever-changing environment.

Ultimately, creating a sense of “we” required that the vision of each community’s Foundry centre be cultivated and maintained by a broad spectrum of stakeholders, including young people and families. In the words of one youth advisor, engaging young people and families who would ultimately access services at the centre was critical for the sense of shared ownership of and responsibility for the centre at the community level. Otherwise, as one youth advisor explained, looking to adult leaders without lived experience of mental health issues would be “like if a vegan was putting together a meat shop”. (Centre C, Youth Advisor)

### “It’s All About Relationships”: Distributed Leadership Promotes Relationship-based Approaches to Service Integration

A significant shift was evident over the course of the evaluation regarding Foundry leaders understanding of how leaders optimally work together to achieve aims of integrated health service delivery. This shift reflects modern scholarship on the nature of complex systems and the importance of relationships within those systems. As Wheatley (2006) describes it, “systems are understood as whole systems, and attention is given to relationships within those networks” [31]. Early accounts from leaders emphasized “partnerships”- which represented formal agreements between organizations to work together in specifically delineated ways (as articulated, for example, in a memorandum of understanding). Once leaders began to work together regularly to address issues that would arise, they began to describe their work together as based in “relationships”- or the deeper but less formal connections established between individuals and organizations.

“It’s about relationship(s) at the end of the day, so, a relational kind of leadership being – at all the different kinds of levels. So, leadership doesn’t only come from my level, but leadership at all the different levels, and relational at all the different levels is crucial.” (Centre D, Staff)

Respondents referred to relationships and relationship-building as active processes that required continuous attention and hard work, particularly when working to transform the highly fragmented and/or siloed contexts in which service providers had been working:

“I think as far as the learning goes, a specific focus on attending to relationship weaves its way throughout this initiative. I think oftentimes – and this has been the experience- like there’s a reason that silos exist because this work is fucking hard.” (Centre A, Staff)

Positive and productive Foundry relationships were characterized by a willingness to find creative approaches to solving problems together, to take shared ownership of identifying and addressing emerging issues and concerns. In the words of one respondent:

“[I]t’s a key part of leadership to be connected to your community, to be connected to your stakeholders, to be connected to potential funders, so when synergies exist that present with critical needs in your community you’re able to respond. I would say that’s been a shift organizationally in our community, but that’s really been about our shift in leadership.” (Centre B, Staff)

Some leaders underscored that the emphasis on building collaborative, trusting relationships to make it easier for leaders to work together across agencies. Systems mirrored what they knew about how best to deliver services to young people:

“It’s all about relationships. You can’t get any work done if the youth don’t respect you in that way or depend on you or know that you’re a safe person.” (Centre E, Staff).

In this respect, the work that was undertaken by leaders to build and strengthen partnerships across sectors and systems was identified as being particularly important for achieving meaningful service integration.

A primary challenge between Lead Agencies and Foundry centres involved internal issues within agencies and disagreements between agencies in the community that remained unresolved when they were brought into Foundry. As one staff member highlighted:

“That philosophical idea at the table of how we’re going to work and then the reality of the different agencies and their restrictions with their policies and procedures and things like that. I think there are still challenges with that and what it means to be not just housed in the same place.” (Centre E, Staff)

Though some of these issues remained unresolved at some centres, distributive leadership allowed for minimizing these challenges, particularly at centres with non-profit Lead Agency partners, through prioritizing youth needs first.

### Streamlining Services towards Integrative Care

As their local centres opened, respondents in all communities spoke to the ways that the distributive approach to leadership had evolved to create their centre was reaping significant tangible benefits, enabling them to implement their approach to integrated care delivery, both at their centres and within the broader community. Distributive leadership approaches enabled a sharing of resources and knowledge between services and sectors, dismantling silos and supporting new partnerships that were necessary for resourcing this new integrated approach to care:

“Yeah, I think some of our best achievements are as we’ve been working towards opening foundry is we’ve already seen increased integration. So we’ve already seen services working closer together because we are meeting so frequently, we are connecting so much more around the foundry initiative. And so it’s also allowing us to connect on – like we have a community of practice that meets monthly and that’s all our frontline staff from all the partners. So there’s already this level of shared knowledge and better referrals and better communication on clients and aspects like that that I think is already improved. And so I think that’s been really exciting.” (Centre D, Staff)

As one respondent emphasized, this also created space for different partners to contribute to this emerging culture of care in ways that allowed partners to play to their respective strengths:

“I think one of the things … is looking at, what are the things that we’re good at? And then, what are the things that other people are good at? So, for example, obviously [FOUNDATION], they’re experts at fundraising. We’re not. So, you take that knowledge of who can do this really well and then you look at opportunity and you put those things together and building a partnership that is of mutual benefit, that’s huge.” (Centre A, Staff)

Through unified and coordinated efforts, access to care was enhanced through implementing drop-in, single point access for youth and families. The availability of drop-in services represented an immediate shift towards service integration in a fragmented system:

“I think the vision that Foundry has is actually being executed really well. So it’s definitely accessible. Like, we’re getting so many clients that haven’t had service before or haven’t been able to access service because they’ve been stuck on different waitlists. So we’ll just have youth walking in, we have parents walk in with the drop-in times… It works really well and people are surprised at how accessible it is.” (Centre E, Staff)

## Discussion

Transforming the health and social care environment for youth and families in British Columbia through Foundry required a tremendous and unprecedented synchronized effort and cooperation. Movement towards distributive leadership as a facilitator for achieving service and system-level integration became increasingly apparent. The following assumptions of distributive leadership were fulfilled to various degrees and led to success. As Foundry continues to expand, distributive leadership shows promise in assuring diverse and coordinated input for integrated service planning and operations.

### Leadership is an emerging key feature of the group

A growth of complexity in health care [32,33] is apparent in settings where team-oriented, integrated health care is pursued [32,34,35]. This was observed during the co-creation of Foundry, where stakeholders faced numerous challenges through evolving processes, and obstacles throughout the transition to create multi-site integrative care centres for youth and families. As noted by Günzel-Jensen et al. 2018, collective representation in leadership activities are essential in such cases. Members belonging to a group develop a shared sense of their capabilities and work together to achieve certain outcomes [36], thereby developing shared social cognitions and psychological ownership [37], which is essential for distributive leadership.

Our findings show distributive leadership emerging through collaborative efforts and relationship building based on a common value system to prioritize youth needs. A prominent theme found in this study was the sense of shared ownership created through the work of Foundry, creating an undercurrent of momentum towards a common goal, which was pivotal for the movement towards distributive leadership.

### Openness towards who can perform leadership tasks, with focus on inclusion rather than exclusion

Through forming partnerships and relationships with key stakeholder groups, leadership structures shifted to expand and accommodate diverse needs from staff and community members alike. Stemming from the sense of ownership over Foundry, the leadership structure “flattened hierarchies”. In addition, reciprocity between Foundry staff and community stakeholders created a dynamic learning environment, ultimately promoting synchronized movement towards a new culture of care. The involvement of youth and family advisory groups in decision-making demonstrate the expansion of the leadership network to involve community in shaping Foundry to fit needs in accordance to site-specific expectations.

### Leadership tasks are shared among the many, not only the appointed leaders

As Foundry sites continued to grow, demands to shift leadership models away from focused leadership was apparent. A sense of “we” created among Foundry stakeholders was critical in creating a platform for distributive leadership, expanding networks to include site-specific leadership tables, stakeholders with diverse professional backgrounds, and community members.

### Limitations

The gathering of findings for this study occurred during nascent stages of Foundry’s development. From stakeholder accounts, we were able to glean the nature of leadership necessary for systems-level integration. Future stages of the developmental evaluation will focus on the perspectives of youth and families accessing services through Foundry to better understand the experience of care delivered through Foundry compared to care as usual, and how these experiences impact clinical outcomes.

## Conclusion

Globally, health services reforms have been implemented to shift away from fragmented provider-centred models of care and reconfigure them around people and communities to ensure that everyone has equitable access to a comprehensive continuum of care [38,39]. Management of complex service innovations, such as integrated health organizations, is demanding and requires a high degree of cooperation between services or integration of services to reduce fragmentation [2]. In the case of Foundry, distributive leadership was effective in promoting streamlined service provision, and coordinating efforts towards optimized access to mental health care services for youth. This transition forged transparency among and between Foundry staff and established community partners. Evidence demonstrated that sharing a youth-focused vision, building trust through collaboration, flexibility within roles, and resource sharing amongst Foundry and community partners were essential for creating a foundational platform for distributive leadership.
